# Dental Trauma at a University Dental Clinic in Milan including the SARS-CoV-2 Period

**DOI:** 10.3390/dj9120145

**Published:** 2021-12-02

**Authors:** Sara Pederielli, Cristina Mirelli, Federico Pozzi, Aldo Bruno Giannì, Roberto Biagi

**Affiliations:** 1 Department of Biomedical Surgical and Dental Sciences, School of Dentistry, University of Milan, Fondazione IRCCS Cà Granda Ospedale Maggiore Policlinico, UOC di Chirurgia Maxillo-Facciale ed Odontostomatologia, Via della Commenda 10, 20122 Milan, Italy; cristina.mirelli@unimi.it (C.M.); aldo.gianni@unimi.it (A.B.G.); roberto.biagi@unimi.it (R.B.); 2 Private Practice of Restorative Dentistry, Via Guglielmo Marconi 2, 20017 Milan, Italy; federico.pozzi54@gmail.com

**Keywords:** dental injury, dental trauma, tooth injury, epidemiology, SARS-CoV-2, COVID pandemic

## Abstract

The aim of this study i.e., is to present the distribution of traumatic dental injuries (TDI) in 306 patients registered at the Unit of Dental Emergencies at a University Dental Clinic in Milan, Italy, between June 2019 and May 2021. This time frame includes the beginning of the SARS-CoV-2 pandemic. Information regarding age, gender, number and type of injured teeth, type of traumatic injury, and data on where or how the injury happened were recorded. Seventy-nine percent of patients can be classified as pediatric (under 14 years old), and in all age groups, male patients were found to be more susceptible (1.6:1). A total of 480 teeth were involved, 59% of which were deciduous, and 41% permanent. The most affected teeth in both dentitions were upper central incisors. In deciduous teeth, periodontal lesions were more common, whereas in permanent dentitions, dental fractures were diagnosed more often. Most data found in this study confirms the results found in the literature. The biggest difference, due to changes in daily routine during the SARS-CoV-2 pandemic, can be found by analyzing the incidence and etiology. As a matter of fact, there was a decrease in school accidents, whereas domestic falls remained constant.

## 1. Introduction

Dental traumatology is a widespread public healthcare problem. It can be influenced by various factors, such as socioeconomic status or environment. Some studies in Europe state that the incidence of TDIs almost doubles in poorer city areas due to less secure school and play-yard infrastructures [1]. Epidemiological reports, however, indicate that most aspects of dental trauma remain relatively consistent. The importance of divulging information regarding this subject lies in its broadness. Epidemiology is particularly interesting for identifying patients’ risk profiles. Prevention is necessary on many levels, and treatment requires a multidisciplinary approach, especially since poorly treated lesions may lead to permanent biological insufficiencies, and psychological or social problems [2,3]. Direct restorations are most commonly used in children, but it is often necessary to reintervene periodically. This leads to a progressive dental tissue loss that may be treated with crowns or veneers [4]. The possible loss of the tooth cannot be treated with implants until the patient is fully developed, requiring the use of alternative types of restauration. Not all of these treatments guarantee a perfect aesthetic, physiological function (phonation, mastication), or the resistance of a natural tooth, and have both a biological and economical cost. Together, all these factors may contribute to the aforementioned problems, such as low self-confidence because of one’s smile, and an inability to talk correctly, and may lead to an unfulfilling social life.

The distribution of dental injuries varies based on age, sex, facial anatomy, hobbies, and handicaps. Most traumatic lesions happen in children, mainly between the ages of 1 and 4 for deciduous teeth, and between 9 and 15 for permanent teeth [5]. During pre-school, domestic accidents and falls are the most common cause of injuries, because children are learning to walk and are very active, but often lack motoric coordination because of their developmental stage [6]. For this reason, they often cannot precisely evaluate velocity and danger. In older children, injuries are more often caused by sports or accidents at school. In middle school they often begin to participate in more intensive sports activities [7,8]. With age, proneness to traumatic dental injuries significantly reduces.

To prevent sports injuries, mouthguards are recommended. They were firstly developed for boxing, and were gradually introduced in other sports thanks to their effectiveness. However, their use may result in discomfort during speech and respiration. Some of these devices are customizable, thereby reducing discomfort, but tend to be more expensive. Whatever the cause, research indicates that mouthguards are not often used. A study reports that even though 80% of the studied population involved in sports activities was aware of the benefits of the mouthguard, only 5% used one [9]. Another study among mountain bikers confirms that many sportsmen do not use such protections as they view them as unnecessary, or find that their breathing is impaired [10]. It is, therefore, extremely important to inform coaches, young athletes, and their parents of the benefits of using protective measures.

Upper central incisors are mostly affected by trauma due to their prominent position. Some conditions, such as 2nd class or labial incompetence, increase the risk of trauma because the superior incisors are even more exposed. The main difference between deciduous and permanent teeth is the type of lesion: periodontal is more common in the first case, and dental structure is more common in the second [11,12,13,14].

The first cases of severe acute respiratory syndrome coronavirus 2 (COVID) were reported in northern Italy in February 2020. To prevent further spread, local emergency restrictions were implemented. On the 8th of March, the Italian government organized a lockdown with school and office closures and strict stay-at-home orders for some regions, including Lombardy. On the 3 June 2020, all travel restrictions were lifted.

A recent study regarding dental health accessibility in Italy reports that, because of the pandemic, only roughly 10% of patients were able to receive medical assistance during the first phase of COVID. Thirty-five percent of interviewed patients avoided seeking medical attention as much as possible for fear of infection, 22% attempted to see a doctor, but the clinics were closed, and 20% did not deem the consultation as necessary [15]. However, the Unit of Dental Emergency at the University Dental Clinic in Milan remained open for critical oral care services, such as TDIs. It can be assumed that some people with minor traumas, such as concussions and enamel infractions, or traumas to deciduous teeth, may have preferred to avoid medical attention due to the fear of contracting COVID.

The aim of this study is to present the distribution of traumatic dental injuries in patients visiting the Unit of Dental Emergency, Department of Biomedical Surgical and Dental Sciences, School of Dentistry, University of Milan, Fondazione IRCCS Cà Granda Ospedale Maggiore Policlinico, UOC di Chirurgia Maxillo-Facciale ed Odontostomatologia, Milan, Italy, from the 1 June 2019 to the 31 May 2021. It is a retrospective epidemiological study designed to evaluate the etiology and the demographic data of the patients included in the study.

## 2. Materials and Methods

This retrospective study includes 306 patients (191 male and 115 female) who were visited in the Unit of Dental Emergency at the Department of Biomedical Surgical and Dental Sciences, School of Dentistry, University of Milan, Fondazione IRCCS Cà Granda Ospedale Maggiore Policlinico, UOC di Chirurgia Maxillo-Facciale ed Odontostomatologia, Milan, Italy, between the 1 June 2019 and the 31 May 2021.

Upon entering the Unit of Dental Emergency, every patient is asked to grant consent to medical treatment and data processing, and, during the COVID pandemic, another consent form for epidemiological COVID data was introduced. Patients are visited in the Unit of Dental Emergencies for immediate response treatment. Based on the lesion, the patient is referred to other units: the Unit of Restorative Dentistry for dental reconstruction and root canal treatment; the Unit of Dental Surgery for element extraction; the Unit of Implantology for implant treatment; and the Unit of Conscious Sedation if the patient in question is not collaborating. Patients presenting themselves with TDI followed the same described standard procedure within the hospital clinic, and were forwarded to the adequate department based on their lesion.

Data for this research was taken from the patients’ clinical records database. No age limit was set, and trauma to both permanent and deciduous teeth were taken into consideration. Patients were divided into age groups (0–4, 5–9, 10–14, 15–19, 20–29, ≥30). Multiple aspects were included in the analysis: basic demographic data; number of patients; age; gender; tooth location; type of dental injury; and etiology. Any missing or unclear information was categorized as unreported.

There are several different classifications for dentoalveolar injuries. Some of them only take anterior teeth into consideration, and some take only tooth injuries. The most complete and, therefore, most commonly used system was applied. It was suggested by the World Health Organization (WHO), and revised by J.O. Andreasen [16]. It includes descriptive classes for dental injuries, periodontal lesions, soft tissue, and bony support system lesions.

The place and/or trauma dynamic was classified into six different categories: home; school; falls; sports and games; traffic accidents; and physical violence.

Data analysis was performed on a Microsoft Excel 365 spreadsheet.

Furthermore, an electronic search of Medline (PubMed), Cochrane, SSCI (Social Citation Index), and SCI (Science Citation Index) databases from 1990 to the present day was performed to collect data on the epidemiology of TDIs. Results of this research were used to compare our findings with historic samples. The following search words were used: tooth injuries; tooth trauma; traumatized teeth; dental trauma; dentoalveolar trauma; oral trauma; epidemiology; etiology; prevalence; dental trauma prevention; risk factors; guidelines; sport trauma. 

## 3. Results

### 3.1. Age and Gender Distribution

**Figure 1:**
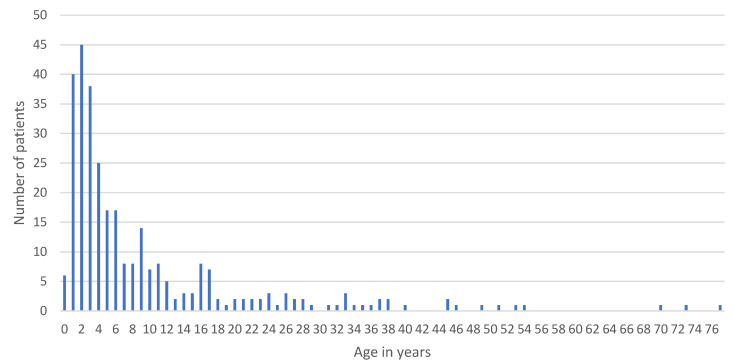
Age distribution.

Three-hundred and six included patients were aged between 0 and 77. If we consider percentage, 79% of patients can be classified as pediatric (under 14 years old), and 50% of all injuries occurred in children under 4 years old, most of which were two-year-old children. Incidence significantly decreases with age (Figure 1).

**Figure 2:**
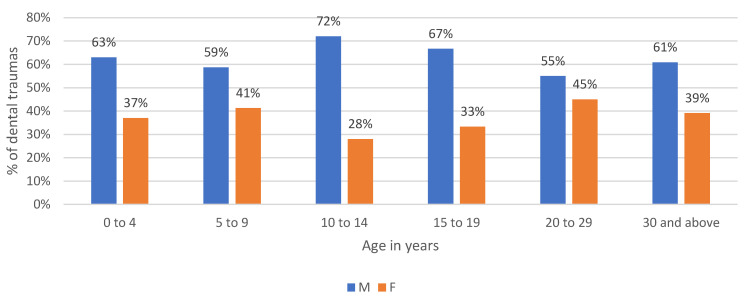
Distribution of traumatic dental injuries in relation to age and gender.

Male/female ratio is 1.6:1. In all age groups, male patients were more commonly affected (Figure 2).

### 3.2. Injuries

Three-hundred and six patients presented dental injuries, with a total of 480 teeth impacted (59% deciduous, 41% permanent); 23 patients had soft tissue wounds, and 4 patients sustained mandibular fractures.

A single tooth fracture was found in 47% of all cases. Children reported less extensive injuries (up to one tooth) more often in comparison to adults (Table 1).

Traumatic dental injuries affecting teeth in the maxilla were significantly more frequent (94%) due to their prominent position.

**Figure 3:**
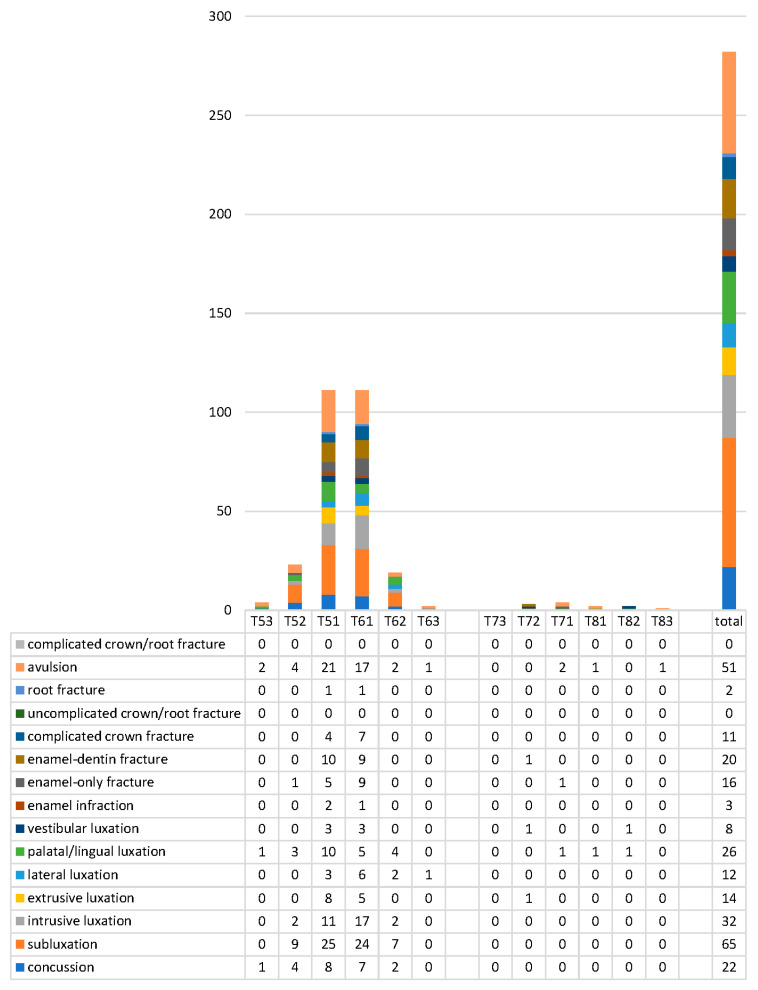
Distribution and types of injuries for deciduous teeth. T53 stands for tooth 53, etc.

**Figure 4:**
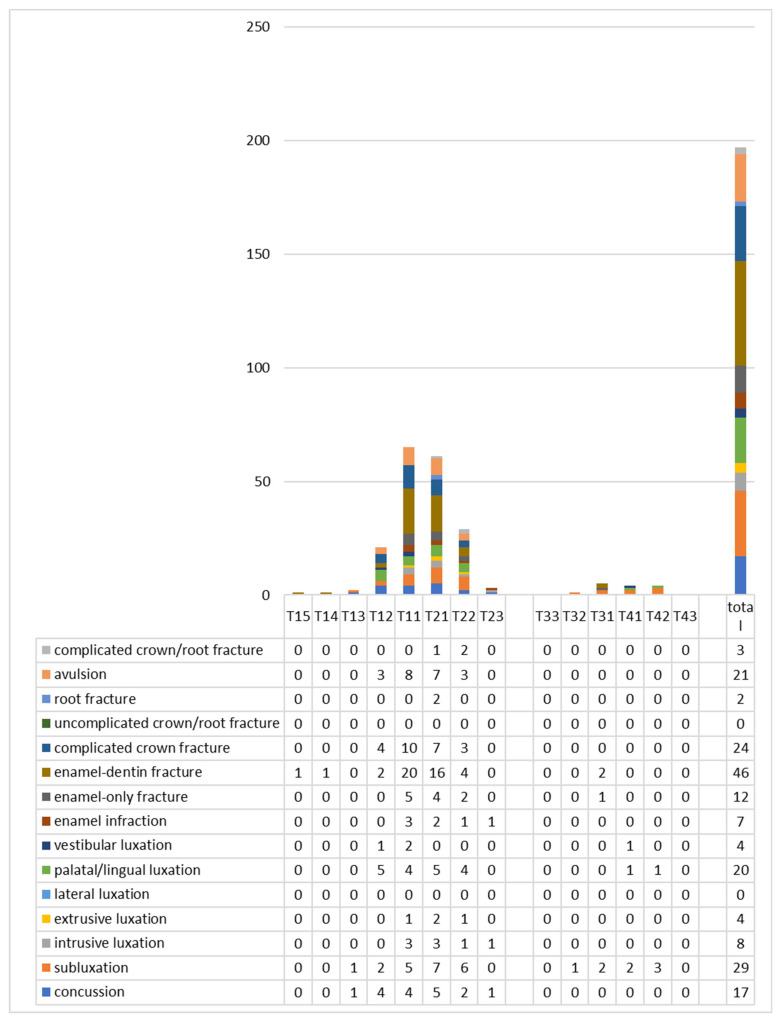
Distribution and types of injuries for permanent teeth. T15 stands for tooth 15, etc.

In both dentitions, maxillary central incisors were mainly involved. In deciduous teeth, right and left central incisors were equally affected (39.4%) (Figure 3), whereas for permanent teeth, 33% of cases involved the right central incisor, and 31% of cases involved the left one (Figure 4). Permanent canines were only concerned in extensive trauma affecting multiple tooth injuries caused by physical violence, or sports or traffic accidents. Only one patient suffered lesions to premolars: the trauma was caused by a bicycle fall in a teenage male patient.

The types of injuries differed between the two dentitions. On one hand, lesions to the periodontal ligament (luxation, 32%; subluxation, 23%; avulsion, 18%) were more common in deciduous teeth (Figure 3.). On the other hand, dental fractures (enamel or enamel and dentin fractures without pulpal involvement, 32%; complicated crown fracture, 12%) were diagnosed in permanent teeth more often (Figure 4).

### 3.3. Etiology of Dental Trauma

**Figure 5:**
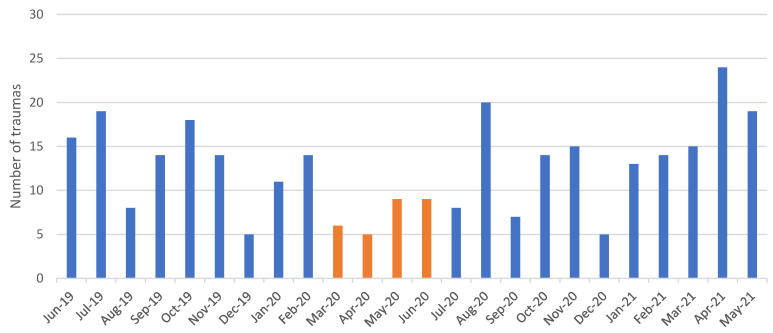
Monthly incidence of TDI. Orange-marked months represent the first main lockdown period.

The monthly distribution showed that during the first months of the COVID pandemic (from March to June), the incidence was reduced due to the stay-at-home order. Other months with significant reduction are August 2019, December 2019, and December 2020, as many people left the city while schools were closed. A peak can be observed during the COVID pandemic in August 2020 since restrictions were lifted, but most people stayed in the city during the holidays (Figure 5).

When the etiology of TDI was analyzed, 13% of medical documentation did not report the etiology. The most common causes of trauma were domestic accidents (40%) and sports injuries (22%). TDIs occurring at school only represented 13%, followed by accidental falls (12%), traffic accidents (9%), and physical violence (4%).

Etiology is very much influenced by the patient’s gender and age. There is a significant difference in the incidence between male and female patients for sports accidents and trauma caused by physical violence. No difference was found for accidental falls (Table 2).

**Figure 6:**
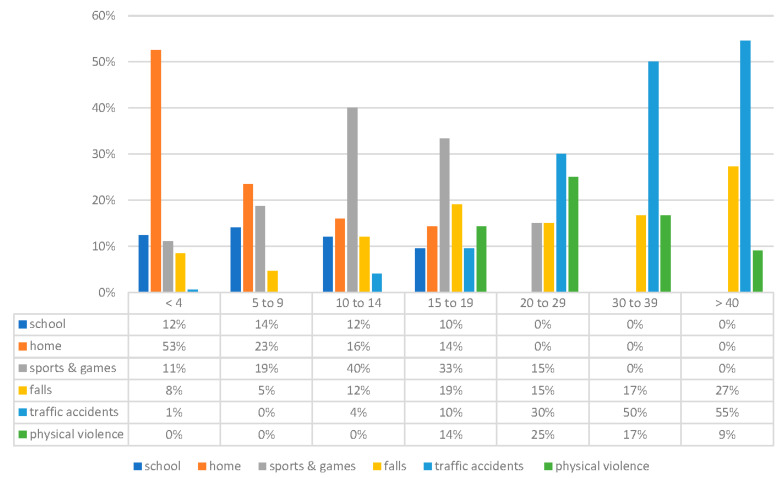
Etiology in relation to age.

As far as age distribution goes, domestic accidents tend to decrease between 0 and 18, whereas sports accidents become the most common cause of trauma between 10 and 14. Physical violence peaks in young adults aged between 20 and 29, and traffic accidents tend to increase with age (Figure 6).

Bicycle falls represented 25% of all sports accidents, and 21% of all traffic accidents. Another 25% of traffic accidents happened on motorbikes. These are important results that could be statistically analyzed if all medical reports specified the sport played when the trauma occurred, or the means of transport and dynamic of the traffic accident.

## 4. Discussion

Since the beginning of the SARS-CoV-2 pandemic, transmission control measures were implemented worldwide. It was the largest attempt at quarantine in human history to prevent the spread of an infectious respiratory disease. The aim of the present study is to evaluate TDI distribution in the general population of Milan (Italy) for a period of two years beginning from June 2019, and compare the data with historic samples published in the literature from previous years. These two years saw the beginning of the pandemic, and transmission control measures greatly influenced certain epidemiological aspects, including the number of patients and etiology. The treatment was not taken into consideration for various reasons. Firstly, many patients were only treated for the emergency, but did not continue treatment at the hospital. Secondly, various types of lesions were treated by different units to guarantee the best specialized care for the patient. Lastly, due to the SARS-CoV-2 pandemic, many follow-up visits were postponed. There are some limitations to this research: it was conducted in a single center, and was, therefore, limited to a subpopulation, mainly Milan’s city center, and data evaluated was recorded by different healthcare professionals, leading to possible bias. Certain minor dental injuries, such as enamel infraction, may not always have been reported, and soft tissue damage may not have been evident if the patients presented themselves after some time had passed since the trauma. Moreover, many patients, especially children, were not always able to recall how the accident happened. Finally, the biggest bias is probably represented by the time-frame (COVID period) in which this study was carried out.

Results regarding the male-to-female ratio (1.6:1), as well as tooth location (upper central incisors), and trauma type (periodontal in primary, and tooth fractures in permanent teeth) are consistent with similar studies [7]. In the literature, sex distribution for dental traumas varies from a male-to-female ratio of 1.28:1 [5] to 2.5:1 [11]. Male patients are usually more commonly affected because they are often involved in more intense sports activities and fights. The exception lies in the pre-school population, where domestic and accidental falls are often similarly distributed, since the risk of falling while learning to walk is the same for both girls and boys [13]. In all studies consulted, maxillary central incisors are always the most affected teeth [1,5,6,7,11,12,13,14]. On average, they represent at least 65% of traumatized teeth, followed by lateral upper incisors, because of their prominent position in the dental arch, especially in some anatomical conditions, such as second class or labial incompetence. Luxation represents the first cause of traumas in deciduous teeth (around 47%) [13]. Conversely, uncomplicated crown fracture is the predominant type of lesion in permanent teeth, representing a percentage ranging between 26.95% [11] and 63.9% [12]. The reasons periodontal tissue injuries are more common in primary dentition are related to the surrounding bone, which is less mineralized, and to the root length that undergoes resorption in order to allow dental exchange, and, therefore, presents less surface for the periodontal ligament.

It is highly possible that the number of minor injuries, especially enamel infractions and concussions, is underestimated. These lesions are often undiagnosed because they are asymptomatic and the patient does not seek treatment immediately. Moreover, some studies report that other minor injuries (uncomplicated crown fractures and lesions involving a single tooth) are more often treated in minor practices in comparison to a hospital clinic [17].

The mean age and age group distribution of patients during the lockdown and other months taken into consideration for this study were similar. Most trauma occurring under the age of 4 were less extensive in comparison to older patients. Children in this age group are more prone to falls at home or around home while learning to walk or playing, due to their developmental stage and their lack of motoric coordination, resulting in less forceful impacts. These are intrinsic risk factors that are not influenced by stay-at-home restrictions. More extensive traumas were observed between the age of 10 and 14 for sports activities, between 20 and 29 for physical violence, and, in adults, for traffic accidents due to the force of the collision. The use of bicycles or motorbikes seems to be an additional risk factor in sports activities and traffic accidents, hence the importance of using a helmet. To prevent sports trauma, the use of a mouthguard should also be encouraged.

The first change can be seen in the frequency of TDIs during the COVID period. When comparing the lockdown period (from March to June) to the previous and the subsequent months, a significant decrease in total number of patients was observed. The reasons behind this vary: the move to indoor life during quarantine diminished the risk of trauma overall [14]. In addition, a recent study regarding dental health accessibility in Italy also reports that only roughly 10% of patients were able to receive medical assistance during the first phase of COVID (from March to June), reducing the overall number of diagnosed cases [15]. Other months that saw a low incidence are August 2019, December 2019, and December 2020, mainly because families left the city while school and extracurricular activities, such as sports, were suspended.

The risk profile of TDIs is closely related to age and gender. Domestic accidents and accidental falls while learning to walk are the primary cause in pre-school children, and affect both boys and girls the same way. School, sports, and games are the most common cause between ages 5 and 14, and affect more male patients [7]. Most falls at school happen during recreation or during physical education lessons. The riskiest sports for TDIs are contact sports, rugby, hockey, American football, and cycling.

Though accidental falls and domestic accidents were scarcely influenced during the pandemic, the number of school mishaps dropped to zero, and traumas due to sports and games went from 19% to 10% because people were quarantined at home without participating in most outdoor activities and sports.

## 5. Conclusions

**Figure 7:**
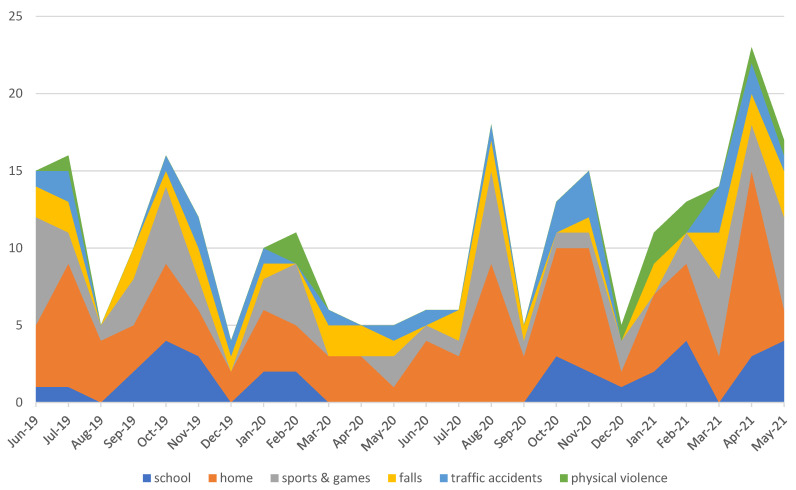
Monthly distribution of etiology.

There are few articles on TDIs during the COVID-19 pandemic [18], resulting in inherent limitations to this study. Additionally, this is a single data collection center, and is, therefore, unable to represent the full scale of TDIs in Milan, Italy. However, results from this study, and those arising when comparing the present data with other published data, demonstrate that the SARS-CoV-2 pandemic had a significant impact on the incidence and etiology of TDIs in Milan (Figure 7). The incidence was reduced by transmission control measures.

Epidemiological research allows us to identify risk groups, for which it is important to implement adequate preventive measures.

Since poorly treated lesions lead to permanent damage, it is important to divulge information regarding TDIs, and the importance of seeking medical attention even if the trauma seems mild. This consideration is always true, especially in these trying times. A handy way to increase public knowledge on this subject is to spread easily accessible information, such as International Association of Dental Traumatology (IADT) posters or their phone application “Tooth SOS”. It explains the easy steps for a correct and immediate response for the general population, and allows healthcare professionals to consult the IADT’s updated guidelines for in-office treatment.

## Figures and Tables

**Table 1:** Number of injured teeth for each patient.

	Deciduous Teeth			Permanent Teeth			
No. of Teeth	Male	Female	Subtotal	Male	Female	Subtotal	Total
1	55	37	92	32	19	51	143
2	39	23	62	22	8	30	92
3	9	6	15	7	7	14	29
4	2	3	5	3	5	8	13
5	0	0	0	0	1	1	1
8	0	0	0	1	0	1	1
Total	105	69	174	65	40	105	279

**Table 2:** Etiology in relation to gender.

	School	Home	Sports and Games	Falls	Traffic Accidents	Physical Violence	Data Not Available
M	60%	57%	79%	50%	63%	91%	55%
F	40%	43%	21%	50%	38%	9%	45%

## Data Availability

The data presented in this study are available in this article.
